# An Edge-Enabled Lightweight LSTM for the Temperature Prediction of Electrical Joints in Low-Voltage Distribution Cabinets

**DOI:** 10.3390/s25226816

**Published:** 2025-11-07

**Authors:** Yuan Gui, Chengdong Yin, Ruoxi Liu, Hanqi Dai, Longfei He, Jiawei Zhao, Quanji Ma, Chongshan Zhong

**Affiliations:** 1 State Gird Beijing Electric Power Research Institute, Beijing 100036, China; pb20253081016@cau.edu.cn (Y.G.); cdyin111@163.com (C.Y.); liuruoxi0121123@163.com (R.L.); dtook@163.com (H.D.); 2 College of Information and Electrical Engineering, China Agricultural University, Beijing 100083, China13948415612@163.com (J.Z.);

**Keywords:** electrical joint, temperature prediction, LSTM, edge computing, lightweight model, low-voltage power grid

## Abstract

Joint overheating in low-voltage distribution cabinets presents a major safety risk, often leading to insulation failure, accelerated aging, and even fires. Conventional threshold-based inspection methods are limited in detecting early temperature evolution and lack predictive capabilities. To address this, a short-term temperature prediction method for electrical joints based on deep learning is proposed. Using a self-developed sensing device and Raspberry Pi edge nodes, multi-source data—including voltage, current, power, and temperature—were collected and preprocessed. Comparative experiments with ARIMA, GRU, and LSTM models demonstrate that the LSTM achieves the highest prediction accuracy, with an RMSE, MAE, and MAPE of 0.26 °C, 0.21 °C, and 0.54%, respectively. Furthermore, a lightweight version of the model was optimized for edge deployment, achieving a comparable accuracy (RMSE = 0.27 °C, MAE = 0.21 °C, MAPE = 0.67%) while reducing the inference latency and memory cost. The model effectively captures temperature fluctuations during 6 h prediction tasks and maintains stability under different cabinet scenarios. These results confirm that the proposed edge-enabled lightweight LSTM model achieves a balanced trade-off between accuracy, real-time performance, and efficiency, providing a feasible technical solution for intelligent temperature monitoring and predictive maintenance in low-voltage distribution systems.

## 1. Introduction

The low-voltage distribution network represents the final stage of electricity delivery to end users, and its failure can directly affect the users. Among its critical components, distribution cabinets play a vital role in allocating load currents among outgoing circuits, where high currents typically flow [1]. Electrical joints in low-voltage distribution cabinets are prone to overheating under complex operating conditions, caused by loose or oxidized connections, load imbalance among phases, and excessive harmonics.

As the current remains steady, the increase in local contact resistance currents results in concentrated Joule heating. The continuous rise in resistance (R) accelerates thermal energy accumulation, rapidly increasing the joint temperature. In severe cases, the temperature can reach a very high level within a short period [2].

Harmonics can also contribute to such heating: on one hand, zero-sequence harmonics (e.g., 3rd, 9th, 15th) superimpose on the neutral line, drastically increasing its current and, thereby, enhancing Joule heating at neutral line joints; on the other hand, the skin and proximity effect induced by high-frequency harmonics raises the AC resistance of electrical joints. Additionally, three-phase imbalance may lead to a dramatic increase in the current of specific lines, causing abnormal Joule heating of the corresponding joints [3,4].

The heat dissipation in a distribution cabinet is generally limited, forming a positive feedback loop that exacerbates the overheating of electrical joints. This may lead to insulation degradation and even sustained smoldering or combustion once the surrounding materials are thermally stressed.

Compared with post-event remediation, short-term temperature-rise prediction based on historical and real-time data provides an actionable early-warning window before risks escalate into failures. This capability has become a key driver for transforming distribution-network maintenance from periodic inspection to condition-based and predictive maintenance [5].

The widespread deployment of online joint temperature monitoring systems and the collection of operational data (e.g., current, ambient temperature, and humidity) in distribution cabinets has enabled the accumulation of long-term multivariate time-series data over multiple time scales, allowing joint temperatures to be predicted from historical information [6].

However, such sequences often exhibit pronounced non-stationarity and multi-scale fluctuations. They are influenced by load switching, ambient temperature and humidity, and equipment heterogeneity. As a result, distributional shifts and abrupt transitions frequently occur, posing significant challenges to conventional statistical models that assume linearity and stationarity [7,8].

In the field of temperature prediction, a variety of time-series modeling approaches have been extensively explored, with the autoregressive integrated moving average (ARIMA) model, the gated recurrent unit (GRU) network, and the long short-term memory (LSTM) network representing the most widely studied methods [9].

In scenarios involving short-term stationary data or sequences with distinct periodic fluctuations, ARIMA offers the advantages of computational simplicity and strong interpretability. However, its predictive capability is substantially limited when confronted with nonlinear fluctuations, abrupt shifts, or multi-scale patterns, as it struggles to capture complex dynamic relationships [10].

The GRU model, as a streamlined variant of the RNN (recurrent neural network), incorporates update and reset gates that reduce parameter complexity while mitigating the vanishing gradient problem. This makes the GRU particularly effective for rapid modeling when dataset sizes are small or computational resources are constrained [11]. Nevertheless, the GRU remains inadequate for capturing long-term dependencies. When applied to highly non-stationary sequences with multiple perturbations, its predictions often lag or underestimate extreme values [12].

In contrast, the LSTM model introduces explicit memory cells and gating mechanisms (input, forget, and output gates), enabling the preservation of historical information over extended time horizons [13]. This structural innovation makes LSTM particularly well-suited for temperature sequences characterized by long-range dependencies, weak periodicity, and superimposed random disturbances—such as the joint temperature rise in distribution cabinets, transformer oil temperature, or the thermal behavior of equipment under varying loads [14]. The empirical evidence consistently shows that the LSTM achieves markedly higher accuracy than both the ARIMA and GRU in forecasting non-stationary and complex time series. For example, studies in transformer oil temperature prediction demonstrate that the LSTM reduces the root mean square error (RMSE) by approximately 60% compared to ARIMA [15], while also outperforming the GRU in capturing rapid transitions and nonlinear upward trends [16].

Since LSTM’s time-series computations involve extensive floating-point operations, the dynamic execution mode of the original TensorFlow framework is inefficient on edge devices without dedicated GPU support. The computational, memory, and power constraints of edge devices at distribution substations make it difficult for full-precision large-parameter deep models to meet the requirements of real-time processing and multi-point concurrent tasks [17]. In a realistic scenario, the vast number of monitored joints in the low-voltage grid produces high-volume data. Centralizing all data processing and prediction in the cloud would impose a heavy burden on communication bandwidth and lead to prohibitive costs [18]. Consequently, it is optimal to perform joint temperature prediction locally at the edge, near the devices.

To address these challenges, this work developed a sensing system based on Bluetooth Mesh IOT to collect multi-source input features (joint temperature, load current, ambient temperature, and humidity) for the prediction models [19]. A Raspberry Pi 5 served as the core of the edge computation device. The LSTM model was trained with data collected from real-world field tests and laboratory experiments. The performance of the LSTM model was evaluated on the electrical joint temperature in a real low-voltage cabinet and was compared with the ARIMA and GRU models. A mixed-precision quantization scheme was applied to optimize the LSTM for lightweight deployment while keeping the network topology unchanged [20]. By comparing the lightweight and original models, the study highlights the differences in model size, inference latency, and prediction accuracy.

This study proposes an edge-enabled lightweight LSTM model for short-term joint temperature prediction in low-voltage distribution cabinets. We demonstrated that this approach achieves superior prediction accuracy compared to traditional methods (ARIMA) and common deep learning models (GRU), while successfully leveraging mixed-precision quantization to ensure millisecond-level inference latency on a Raspberry Pi 5 edge device. The findings validate a practical and cost-effective technical pathway for implementing real-time predictive maintenance in large-scale low-voltage distribution networks.

Although LSTM-based models have been extensively investigated in various domains such as energy forecasting, machinery fault detection, and climate prediction, no previous study has reported the application of an edge-enabled LSTM for predicting the temperature of electrical joints in low-voltage distribution cabinets. In practical power distribution systems, the very large number of joints makes centralized temperature monitoring and analysis impractical. Therefore, developing an edge-based AI prediction model represents a necessary and novel approach, enabling localized data processing and predictive analytics directly at the cabinet level. This study thus provides a new perspective for implementing intelligent, distributed, and real-time monitoring in power systems.

The remainder of this paper is structured as follows: Section 2 outlines the materials and methods, including the sensing system and data acquisition, the LSTM model methodology, and the lightweight optimization scheme; Section 3 presents the experimental results and comparative analysis; and Section 4 and Section 5 provide the discussion and conclusions.

## 2. Materials and Methods

### 2.1. Mathematical Formulation of the Time-Series Prediction Problem

Mathematically, joint temperature forecasting can be abstracted as a multi-step time-series forecasting task. Let the historical observation sequence be

(1) $$X_{t}=\{x_{t-L+1},x_{t-L+2},\ldots ,x_{t}\},$$

where $x_{i}\in R^{d}$ represents the d-dimensional input feature vector at time step *i*, including electrical quantities (voltage, current, power), environmental quantities (temperature, humidity), and historical joint temperature. The prediction target is to obtain the joint temperature for the next H steps

(2) $${\hat{Y}}_{t,h}=\{{\hat{y}}_{t+1},{\hat{y}}_{t+2},\ldots ,{\hat{y}}_{t+H}\},$$

where ${\hat{y}}_{t+k}$ denotes the predicted joint temperature at the k-th future time step.

To quantify the discrepancy between the predictions and the ground truth, commonly used metrics include the root mean square error (RMSE), mean absolute error (MAE), and mean absolute percentage error (MAPE):

(3) $$RMSE=\sqrt{\frac{1}{N}\sum_{i=1}^{N}{\left(y_{i}-{\hat{y}}_{i}\right)}^{2}},$$

(4) $$MAE=\frac{1}{N}\sum_{i=1}^{N}|y_{i}-{\hat{y}}_{i}|,$$

(5) $$MAPE=\frac{100\%}{H}\sum_{k=1}^{H}\left|\frac{y_{t+k}-{\hat{y}}_{t+k}}{y_{t+k}}\right|,$$

where $y_{t+k}$ represents the true observed value. Through these indicators, the performance of the prediction model in terms of accuracy and stability can be comprehensively evaluated.

### 2.2. Prediction System Architecture

**Figure 1:**
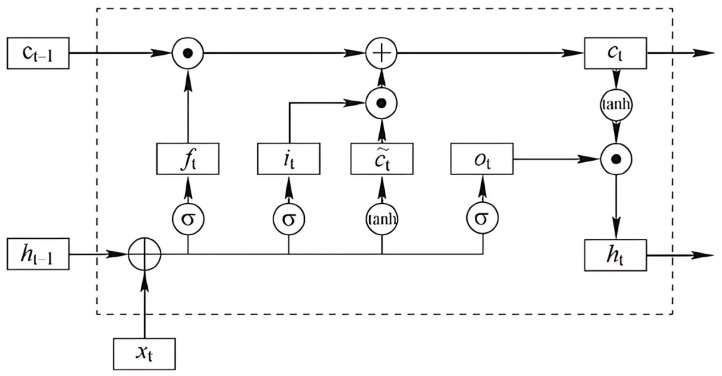
LSTM architecture.

An LSTM network comprises three key components—the input gate, forget gate, and output gate—which, respectively, regulate the inflow, retention (memory), and outflow of information, as illustrated in Figure 1.

The node outputs of the LSTM network are computed as follows:

(6) $$f_{t}=\sigma (W_{f}\cdot [h_{t-1},x_{t}]+b_{f}),$$

(7) $$i_{t}=\sigma (w_{t}\cdot [h_{t-1},x_{t}]+b_{i}),$$

(8) $${\tilde{c}}_{t}=\tanh(W_{c}\cdot [h_{t-1},x_{t}]+b_{c}),$$

(9) $$C_{t}=f_{t}\cdot C_{t-1}+i_{t}\cdot {\tilde{c}}_{t},$$

(10) $$h_{t}=o_{t}\cdot \tanh(C_{t}).$$

In the above equations, $x_{t}$ denotes the input feature vector at time step t, while $h_{t}$ and $h_{t-1}$ represent the hidden states at the previous and current time steps, respectively. The cell state *C_t_* serves as the long-term memory that carries information across time steps, with *C_t−_*_1_ denoting its value from the previous step. The forget gate *f_t_* determines the extent to which information from *C_t−_*_1_ is retained, the input gate $i_{t}$ controls the incorporation of new information from the candidate cell state ${\tilde{C}}_{t}$, and the output gate $o_{t}$ regulates the portion of the cell state that contributes to the hidden state output. The candidate cell state ${\tilde{C}}_{t}$ represents the new information proposed to be added to the memory cell. Matrices *W_f_, W_t_, W_i_, Wc*, and W_0_ and bias terms *b_f_, b_t_, b_i_,* and *bc* correspond to the learnable parameters associated with each gate. The nonlinear activation functions σ(.) and *tanh*(*∙*) ensure that the gating operations and state updates remain bounded within appropriate ranges.

To further alleviate the cold-start error caused by random initialization, this work introduced a warm-start strategy based on power-difference matching [21].

First, the power-difference vector is defined as Δ*x_t_ =* [Δ*P_t_*, Δ*S_t_*]. Here, Δ*P_t_* = *P_t_ − P_t−_*_1_, and Δ*S_t_* = *S_t_* − *S_t−_*_1_, where *P_t_* and *S_t_* denote the active power and apparent power at time step t, respectively. By employing a similarity measure (e.g., Euclidean distance or cosine similarity), the most similar historical segment is retrieved from the set of past operating conditions {X^(*i*)^}. The optimal index is defined as

(11) $$i^{*}=\arg\min_{i}dist(\Delta x_{t},\Delta x^{\left(i\right)}),$$

and the corresponding reference segment is expressed as

(12) $$X^{\left(ref\right)}=X^{\left(i^{*}\right)}.$$

Finally, the hidden state at the last time step of the reference segment $\left(h_{T}^{\left(ref\right)},c_{T}^{\left(ref\right)}\right)$ is adopted as the initial hidden state of the current prediction window:

(13) $$(h_{0},c_{0})=(h_{T}^{\left(ref\right)},c_{T}^{\left(ref\right)}).$$

Compared with the conventional random initialization (*h*^0^,*c*_0_)~*N*(0,*σ*^2^), the proposed method transfers hidden state information from similar operating conditions, thereby enhancing the temporal alignment and prediction accuracy in peak–valley neighborhoods and rapid transition periods.

In this study, the application of the LSTM network not only effectively addresses the challenge of modeling long-term sequential dependencies in joint temperature prediction but also ensures training convergence and predictive robustness [22]. Through its gating mechanisms, the LSTM dynamically adjusts the retention or forgetting of historical information in response to changes in the input signals, thereby accommodating non-stationary factors—such as current fluctuations and periodic variations in ambient temperature and humidity—that induce fluctuations in the temperature-rise trend.

### 2.3. Model Training Settings

All deep learning models were implemented in Python 3.10 using the PyTorch 1.10 framework. The LSTM, GRU, and ARIMA baselines were trained under the same data-splitting strategy (70% training, 15% validation, and 15% testing). For LSTM and GRU, the sequence length L was set to 60 time steps, and the prediction horizon H was defined as 6 h, unless otherwise stated.

The model parameters were optimized using the Adam optimizer with an initial learning rate of 0.001, and the mean square error (MSE) loss was adopted as the training objective. A batch size of 64 was used to balance the memory efficiency and convergence stability. Early stopping with a patience of 10 epochs was applied to avoid overfitting, and the maximum number of training epochs was set to 200.

To prevent data leakage and maintain experimental integrity, the dataset was first divided into training and testing subsets before normalization. The normalization parameters (minimum and maximum values) were calculated exclusively from the training data and then applied to the validation and testing sets using the same scaling transformation. During model evaluation, the predicted temperature values were inverse-transformed to restore their original physical scale prior to error computation, ensuring the comparability and interpretability of the RMSE, MAE, and MAPE metrics.

To incorporate the proposed warm-start mechanism, hidden and cell states were initialized using the retrieved reference states rather than random values, enabling faster convergence and reducing the cold-start prediction errors. All experiments were conducted on an NVIDIA RTX-series GPU server with 24 GB memory, ensuring efficient training and inference.

Compared with conventional random initialization, the proposed warm-start initialization accelerated the convergence by approximately 20% and reduced the early-stage prediction error by around 10%. This improvement was achieved by transferring hidden-state information from similar operating conditions, providing the network with a more suitable temporal context at the beginning of training. To ensure optimal training stability, the key hyperparameters—including the sequence length, learning rate, and batch size—were tuned using a grid-search strategy on the validation set. Within a ±10% variation range of these parameters, the model’s RMSE fluctuation remained below 3%, demonstrating that the warm-start strategy effectively enhanced both the convergence efficiency and prediction robustness.

### 2.4. Data Acquisition

**Figure 4:**
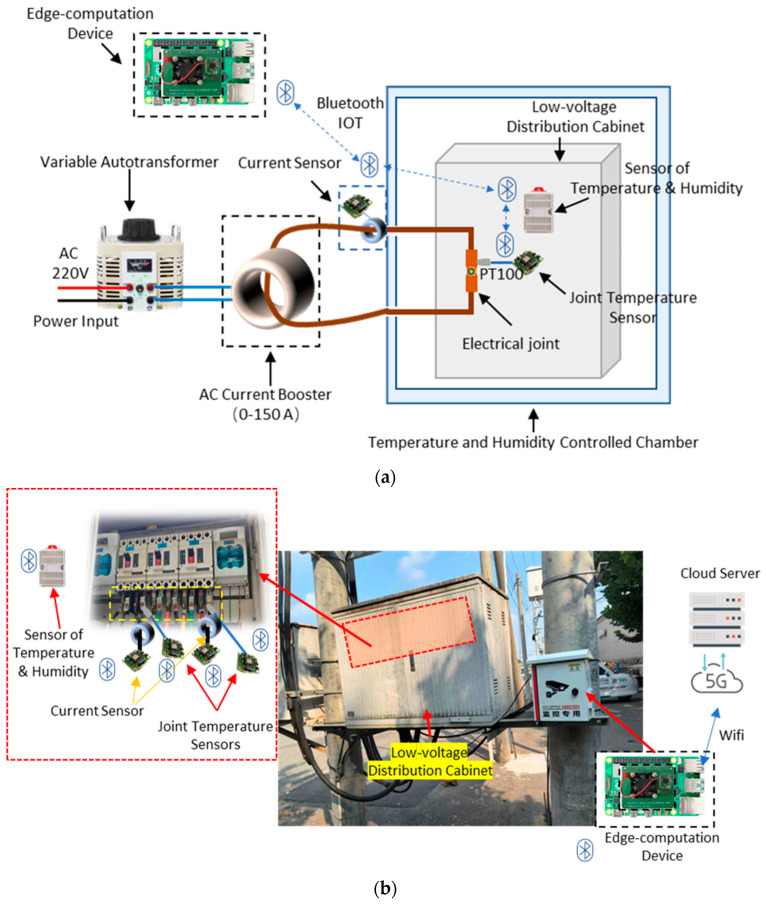
Schematic diagram of experimental scenario. (**a**) Experimental setup in laboratory; (**b**) Experimental setup in real low-voltage distribution cabinet.

Due to the scarcity of joint overheating and fault samples in real operating data, the data for training and testing the models were acquired from both real low-voltage and laboratory cabinets. To assess the predictive performance of the model under extreme working conditions, a simulated heating fault platform for distribution cabinet joints was designed and constructed in the laboratory, as shown in Figure 4a. This platform employed systems for adjusting the contact resistance and load current to generate abnormal joint states of varying severity, thereby reproducing the temporal joint temperature rise and the corresponding fault development process. The real-world experimental setup is displayed in Figure 4b, where the cabinet is installed near a 10 kV/400 V transformer.

The experimental principle was based on the heating mechanism of contact resistance: when the surface roughness, oxide film thickness, or clamping force of the contact changes, the contact resistance $R_{c}$ will increase, causing the current flowing through the joint to generate additional Joule heat Q:

(14) $$Q=I^{2}Rt,$$

where *I* represents the RMS value of the current through the joints, while *t* is the energization time. When $R_{c}$ increases to a certain extent, the local temperature of the joint will continue to rise and may cause thermal instability. In the experiment, by fine-tuning the clamping force and contaminating the contact surface, $R_{c}$ is increased in stages within a safe range, thereby precisely controlling the temperature rise rate and peak value.

As illustrated in Figure 4a,b, the arrangement was as follows. The simulated joint module was placed at the center of the distribution cabinet, while the current regulation module was connected in series via low-resistance conductors to adjust the current. The joint temperature measurement module was affixed directly to the surface of the joint to monitor its temperature, and the ambient temperature and humidity measurement module was in the middle of the cabinet to record the internal environmental conditions. The current acquisition module was employed to measure the cable current. All data acquisition modules transmit signals via a Bluetooth Mesh network to a Bluetooth client module, which then forwards the data via the Raspberry Pi’s hardware serial port to the data collection module [23].

A Bluetooth Mesh-based wireless-sensor network was developed for this work, consisting of multiple sensor nodes and a central client receiver. Each joint temperature sensor includes a PT100 platinum temperature probe (−50 °C to 250 °C, accuracy ±0.2 °C), an nRF52832 Bluetooth Mesh module, and a 3.7 V lithium battery for power supply. The nodes are installed at electrical–joint interfaces and secured with thermally conductive adhesive to ensure accurate heat coupling and reliable measurements.

**Figure 2:**
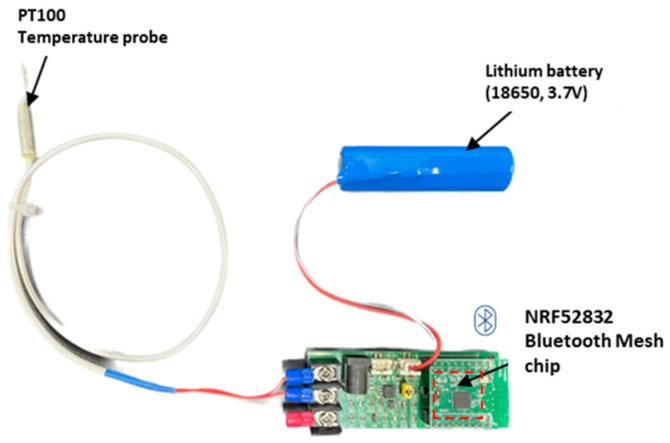
Physical module of the temperature sensing node.

**Figure 3:**
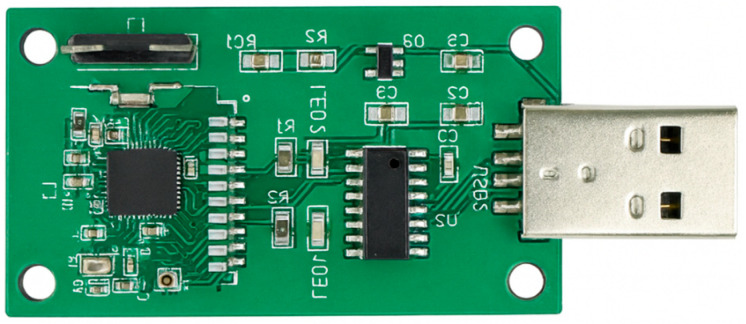
Physical module of the Bluetooth Mesh client receiver.

The Bluetooth Mesh client receiver acts as a gateway that collects temperature data from all nodes and forwards the data to the edge server through a USB interface. The hardware modules and PCB layouts are shown in Figure 2 and Figure 3, respectively, illustrating the practical implementation of the temperature monitoring system for low-voltage distribution cabinets.

The self-developed sensors used to monitor the ambient temperature, humidity, and load current (20–200A) are shown in Figure S1 and Figure S2, respectively.

In the setup, the current acquisition module, current regulation module, and data collection module are positioned outside the temperature–humidity control enclosure. The conductors enter the enclosure through feedthrough holes in its outer wall. All other components are housed inside the temperature–humidity control enclosure.

In total, the monitoring system continuously collected joint temperature data for more than 15 days under both field and laboratory conditions, with a 3 min sampling interval. Each distribution cabinet included multiple measurement points, yielding over 20,000 valid temperature records following data preprocessing. This dataset effectively captured both short-term fluctuations and long-term heating trends across varying loads and ambient temperature and humidity in the cabinet. For model training, a sliding-window approach was adopted: each input sequence consisted of 60 consecutive time steps (corresponding to 3 h of historical data), which were used to predict the temperature evolution over the subsequent 6 h horizon. This configuration not only provided sufficient temporal variability to support model learning but also aligned with the practical time resolution requirements of edge devices.

Under the arrangement, graded contact resistance and load condition tests were conducted, yielding temperature time-series data covering the stages from normal operation to mild abnormality and further to severe abnormality/fault. Representative joint temperature-rise curves and stage divisions are shown in Figure 4. It can be observed that, during abnormal stages, the curves exhibit pronounced nonlinear increases accompanied by multi-scale fluctuations. Near peak values, the temperature-rise rate increases markedly. Following the activation of protective actions or load mitigation measures, the temperature drops rapidly and transitions into a stable stage. This behavior shows good consistency with the field observations and provides a reliable basis for subsequent model training and extreme condition evaluation.

The dataset used in this study was collected through the same data acquisition system developed by our research group, consisting of two complementary subsets: a field dataset from outdoor low-voltage distribution cabinets in a district of Beijing, reflecting real operating conditions, and a laboratory dataset obtained under controlled fault-heating experiments. Both subsets shared identical sensing hardware, data acquisition protocols, and edge communication architecture (Bluetooth Mesh to Raspberry Pi gateway), ensuring consistency in format and quality. Data were recorded every 3 min from multiple measurement points for approximately 15 consecutive days. After preprocessing, more than 20,000 valid samples were obtained. To ensure reliable model training and evaluation, the dataset was divided chronologically into 70% for training and 30% for testing, maintaining the temporal integrity of the time series. Each data record contains the voltage, current, power, joint temperature, and ambient temperature variables. All features were normalized using the min–max method, and the model’s predictions were inverse-transformed before calculating the RMSE, MAE, and MAPE to ensure that the results retained their physical interpretability. Each training sample consists of 60 consecutive time steps (3 h of historical data) used to predict the following 6 h temperature evolution. This unified configuration enhances the comparability between the field and laboratory datasets and ensures a coherent and physically meaningful model input structure.

### 2.5. Data Preprocessing

The same preprocessing procedures were applied to both the field and laboratory datasets to ensure data consistency. Specifically, the field dataset, collected from outdoor distribution cabinets in a district of Beijing, represents real operating conditions, while the laboratory dataset provides controlled abnormal-heating sequences for model robustness verification. The preprocessing steps included missing-value interpolation, outlier correction using the Z-score method, and min–max normalization, which together ensured the integrity and uniformity of the dataset.

The dataset employed in this study was collected from outdoor distribution cabinets located in a specific district of Beijing, China, recording the dynamic variations in joint temperature during actual operation. Due to the complexity of the monitoring environment, the raw data inevitably contained missing points, outliers, and noise. If such data were fed directly into the prediction model without cleansing, the training process would likely become unstable, and the predictive accuracy could be degraded [24]. Therefore, prior to modeling, systematic data preprocessing was required to ensure the reliability and stability of the input data.

For missing-value handling, linear interpolation was employed to impute the temperature time series. The underlying principle was to estimate each missing value using the two adjacent valid observations immediately preceding and following it:

(15) $$x_{t}=x_{t-1}+\frac{x_{t-1}-x_{t-1}}{\left(t+1)-(t-1\right)}\cdot (t-(t-1)).$$

**Figure 5:**
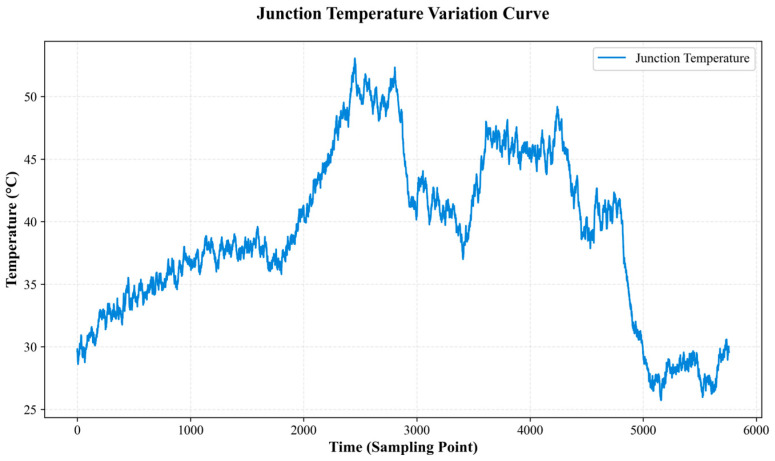
Simulation joint heating experiment curve chart.

This method filled the gaps while preserving the continuity of the overall trend. As shown in Figure 5, the imputed sequence became smoother, preventing abrupt interruptions at the model input.

**Figure 6:**
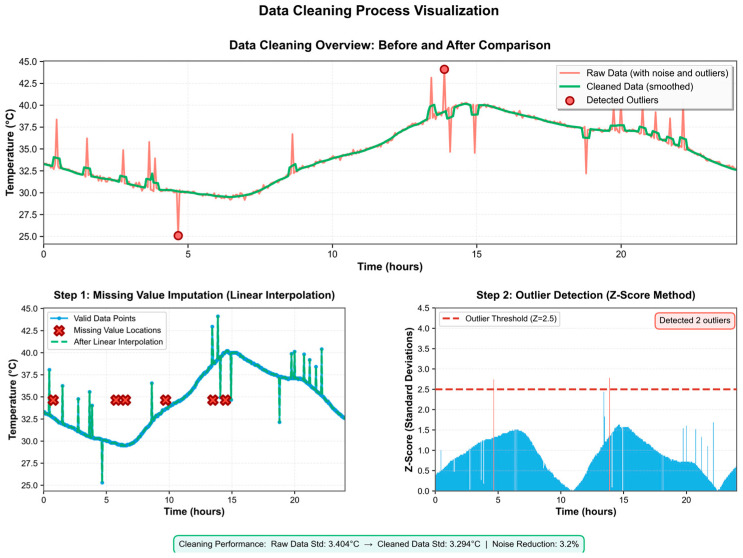
Data cleaning diagram.

For outlier detection, the Z-Score method was introduced to identify and correct spikes or abrupt changes. The specific computation was given by

(16) $$Z_{i}=\frac{x_{i}-\mu }{\sigma }$$

where μ and σ are the mean and standard deviation of the sequence, respectively. When $\left|Z_{i}\right|$ > 2.5, this point is determined as an outlier and replaced by the smoothed value at adjacent moments. In this way, the abnormal sharp peaks caused by the instantaneous interference of the sensor are effectively removed. As shown in Figure 6, the processed sequence significantly reduces extreme abrupt changes, and the overall curve is more continuous.

Finally, to avoid the adverse effects of differing feature scales on model training, all the input variables—including voltage, current, humidity, and temperature—were normalized using the min–max scaling method:

(17) $$x_{i}^{\prime }=\frac{x_{i}-\min(x)}{\max(x)-\min(x)}.$$

The data were scaled to the [0,1] range. This process not only improved the numerical stability during training but also accelerated the model convergence. As shown by the overall comparison in Figure 6, the sequence becomes smoother and more stable after missing-value imputation, outlier correction, and normalization, thereby providing high-quality inputs to the LSTM model.

After preprocessing, the data served as the final dataset for model training and subsequent prediction tasks.

### 2.6. Lightweight Model of LSTM

When deploying a full-precision (FP32) LSTM model on edge platforms such as the Raspberry Pi, the ARM Cortex-A72 quad-core CPU (without a dedicated GPU), along with the limited memory and bandwidth, often fails to meet the real-time constraints of online prediction due to inference latency and resource consumption. To reduce the computational and storage overhead while preserving predictive accuracy as much as possible, a mixed precision quantization scheme was adopted to lightweight the trained model.

Specifically, 8-bit integer (INT8) quantization was applied to the storage of weights and selected activations to compress the model size and memory access cost, half-precision floating point (FP16) was used in key matrix multiplication operations to maintain numerical stability and throughput, and INT8 operators were employed for non-critical path computations to further reduce latency. This scheme required no changes to the LSTM network topology, modifying only the numerical representation and operator precision, thereby facilitating stable deployment on resource-constrained devices.

Quantization and dequantization adopt affine mapping. Let the original floating-point tensor (weight or activation) be ω*_f_*, the quantized integer tensor be ω*_q_*, the scaling factor be *S*, and the zero point be *Z*. Then, the relationship between quantization and dequantization is

(18) $$\omega_{q}=round(\frac{\omega_{f}}{S})+Z,$$

(19) $$\omega_{f}\approx S\cdot (\omega_{q}-Z),$$

where the scaling factor (*S*) and zero (*Z*) point were given by the upper and lower bounds of the quantization interval:

(20) $$S=\frac{\omega_{f,\max}-\omega_{f,\min}}{q_{\max}-q_{\min}},$$

(21) $$Z=round(q_{\min}-\frac{\omega_{f,\min}}{S}).$$

We perform boundary clipping during implementation to avoid integer overflow or saturation:

(22) $$\omega_{q}=clip(round(\frac{\omega_{f}}{S})+Z,q_{\min},q_{\max}).$$

Among them, for INT8, *q_max_*= 127 and *q_min_*= −128 are usually taken to balance the precision and inference efficiency. Weight quantization adopts a per-channel configuration; that is, *S_k_* and Z*_k_* are independently estimated for each output channel *k* to suppress the quantization errors caused by channel–scale differences; activation quantization adopts a per-tensor configuration to simplify the inference path and reduce the overhead of boundary de-quantization. In the calibration phase, statistics of the validation data (such as min/max or quantile clipping) were used to estimate [*ω_f,min_*, *ω_f,max_*], and *S* and *Z* were selected with the criterion of minimizing the quantization errors (such as MSE/MAE); when the quantization sensitivity of individual layers is relatively high, a small number of steps of Quantization-Aware Training (QAT) can be used for fine-tuning to recover the precision loss.

During inference, integer cores and floating-point boundary operators co-ordinate. Taking the main multiply–accumulate operations in the LSTM gates (i, f, o, g) as an example, the inputs and weights were quantized and accumulated within the integer domain:

(23) $$y_{\mathrm{int}32}=\sum_{j}(W_{q}^{\left(j\right)}-Z_{W}^{\left(j\right)})\cdot (x_{q}^{\left(j\right)}-Z_{x}).$$

The layer outputs are dequantized at the boundaries and passed into the nonlinear operators:

(24) $$\hat{y}=\sigma (y_{fp}^{\left(i\right)})⊙\tanh(y_{fp}^{\left(g\right)}).$$

In this process, σ(∙) and tanh(∙) were executed in the FP16/FP32 domain to ensure numerical stability, while accumulation was performed in the INT32 domain to avoid overflow. Subsequently, a combination of scaling factors was applied to complete the mapping from the integer domain to the floating-point domain. An upper bound can be given for the discrete approximation error introduced by affine quantization:

(25) $$\omega_{f}-S\cdot (\omega_{q}-Z)|\leq \frac{S}{2}$$

Smaller scaling factors (i.e., tighter dynamic ranges) reduce the maximum error of a single quantization step; however, an overly small range increases the risk of saturation/clipping, requiring a calibration-stage trade-off between the approximation error and saturation probability. Overall, the mixed-precision scheme achieved FP32-to-INT8 compression at the parameter level and FP32-to-FP16/INT8 precision reduction at the operator level, substantially reducing the model size, memory bandwidth requirements, and end-to-end inference latency, while maintaining floating-point precision in key nonlinear operations to suppress numerical instability.

## 3. Results

### 3.1. Analysis of Testing Set Performance

This section evaluates the performance of the proposed predictive model on an independent testing set. The testing data were collected from real-time operational records of multiple low-voltage distribution cabinets in a district of Beijing and were subjected to the same data preprocessing and normalization procedures as in the training phase, to ensure the comparability and consistency of the results.

**Figure 7:**
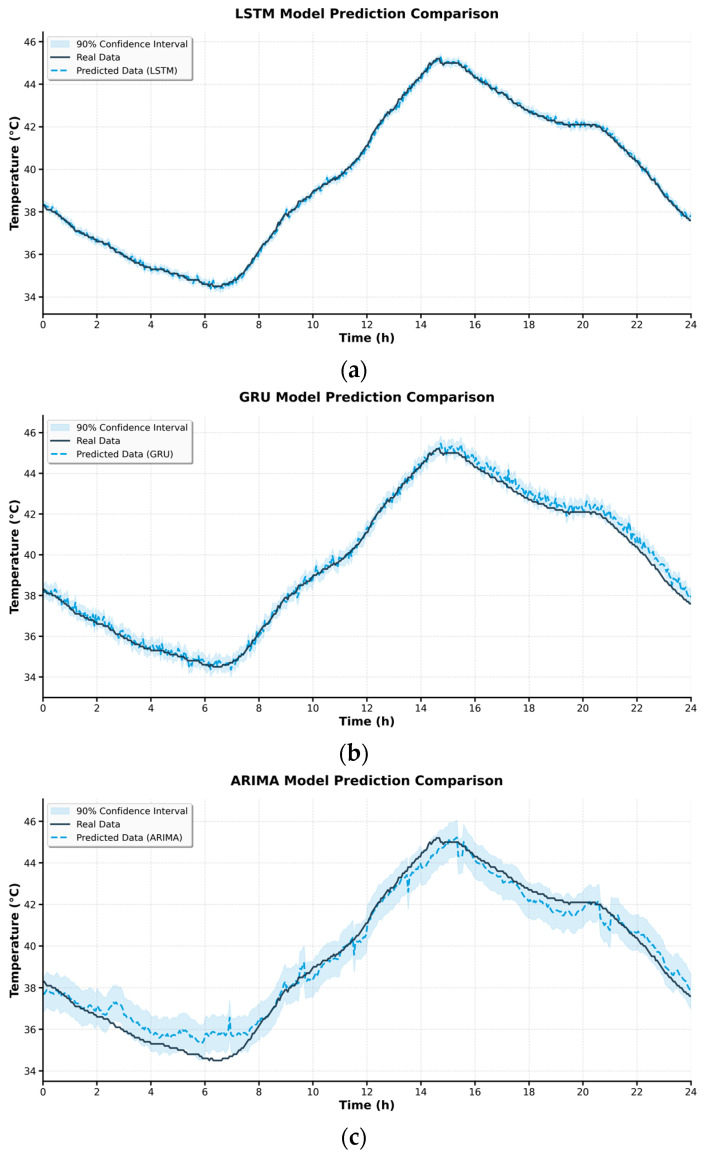
Comparison of the fitting effects of different models on the joint temperature data in the testing set. (**a**) LSTM Model Prediction Comparison; (**b**) GRU Model Prediction Comparison; (**c**) ARIMA Model Prediction Comparison.

As shown in Figure 7, the temperature fitting results of the real measurements and the three models (ARIMA, GRU, and LSTM) on the testing set are presented. The original three fitting curves were merged into a single plot with unified time axes and coordinate scales. Enlarged windows were included for key intervals to facilitate analysis of the models’ performance during peak, trough, and rapid-change stages.

It can be observed that the LSTM curve outperformed the other models in both temporal alignment with temperature variations and amplitude accuracy, exhibiting smaller residuals in peak segments. The GRU model performed well in most steady-state intervals but showed a certain response lag in high-frequency fluctuation periods. The ARIMA model depicted the overall trend in a relatively smooth manner; however, its deviation increased significantly in non-stationary intervals, especially near sharp peaks. This is consistent with the known limitations of traditional linear modeling frameworks in handling complex nonlinear time-series data.

The ARIMA model exhibited larger deviations during non-stationary intervals with rapid temperature fluctuations, primarily due to its linear modeling assumption, which limits its ability to capture nonlinear and time-varying dependencies in joint temperature evolution. These findings confirm that the proposed LSTM model provides more stable and accurate predictions under real field conditions.

**Figure 8:**
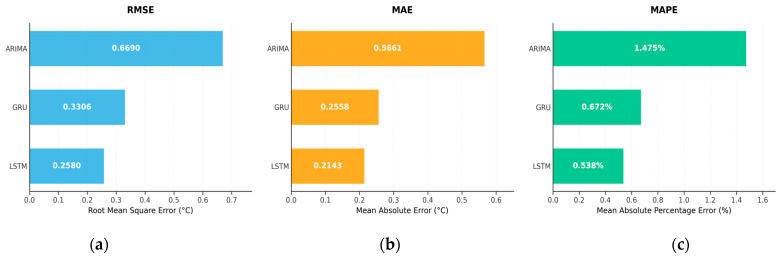
Comparison of the error metrics for the testing sets. (**a**) RMSE error comparison chart; (**b**) MAE error comparison chart; (**c**) MAPE error comparison chart.

To quantitatively assess the aforementioned fitting differences, the root mean square error (RMSE), mean absolute error (MAE), and mean absolute percentage error (MAPE) were calculated, with the results shown in Figure 8. The ranking of these three metrics remained consistent across the models: the LSTM model achieved the lowest RMSE, MAE, and MAPE on the testing set, followed by the GRU, while the ARIMA yielded the highest values. Compared with the LSTM, the GRU showed only a limited performance drop, whereas the ARIMA exhibited a substantial increase in error for all metrics. These results indicate that deep networks incorporating long-term dependency modeling and nonlinear feature learning offer significant advantages in capturing complex temperature evolution patterns and mitigating prediction bias.

### 3.2. Evaluation of Online Deployment and Generalization Performance

#### 3.2.1. Multi-Horizon Forecasting for the Same Distribution Cabinet

In the online deployment scenario for the same distribution cabinet joint, the LSTM model was evaluated under three forecast horizons (6 h, 24 h, and 3 days) to assess the short- and long-term predictive accuracy.

**Figure 9:**
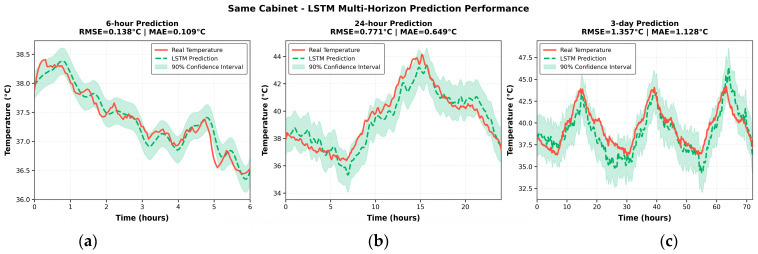
LSTM multi-horizon temperature prediction performance for the same distribution cabinet; (**a**) LSTM 6-h Temperature Prediction for the Same Cabinet; (**b**) LSTM 24-h Temperature Prediction for the Same Cabinet; (**c**) LSTM 3-Day Temperature Prediction for the Same Cabinet.

As shown in Figure 9, the 6 h forecast closely matches the measured temperature curve in both peak and trough positions, as well as in rates of change. The response remains smooth with minimal delay, and the residual band is narrow and nearly uniform across most time windows.

For the 24 h forecast, the model maintains good tracking of long-term trends, although slight amplitude underestimation appears near peaks in non-stationary intervals, and mild over-smoothing occurs at troughs. For the 3-day forecast, the model still captures the principal trend but exhibits increased uncertainty, reflecting error accumulation and distributional drift in long-horizon forecasting.

**Figure 11:**
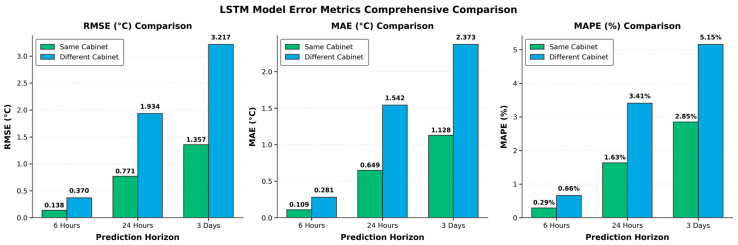
Comparison of prediction error metrics under two deployment scenarios.

For all three horizons, the same LSTM model structure and data preprocessing steps were used to maintain comparability. The output windows were set to 6, 24, and 72 time steps, respectively, while the 24 h and 3 d predictions were generated directly, avoiding iterative accumulation of short-term results. As illustrated in Figure 11, the RMSE values for the 6 h, 24 h, and 3 d forecasts were 0.83 °C, 1.17 °C, and 1.64 °C, respectively, indicating reliable temporal consistency and accuracy across different horizons.

#### 3.2.2. Cross-Cabinet Generalization Analysis

To evaluate generalization capability, the model was deployed across different distribution cabinets with varying hardware and environmental conditions.

**Figure 10:**
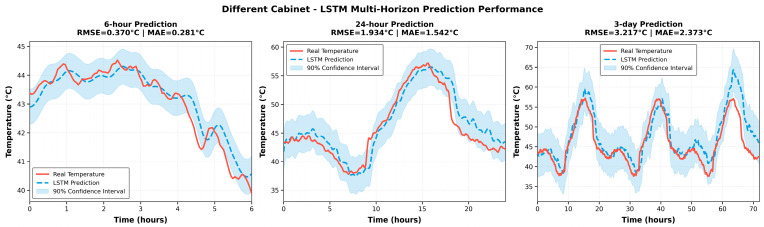
Temperature prediction fitting results under different distribution cabinet joint scenarios.

As shown in Figure 10, the 6 h forecast maintains good phase alignment and amplitude accuracy, comparable to the same-cabinet case. However, with an increasing forecast horizon (24 h and 3 days), the deviations become more evident: the peak amplitudes slightly decrease, response lags appear on rising edges, and uncertainty intervals widen.

In the 3-day forecast, these deviations are further amplified, reflecting the combined effects of load imbalance, cabinet aging, and ambient temperature differences. Nevertheless, the model preserves the correctness of the long-term trends without systematic drift, confirming its applicability under unseen cabinet configurations.

#### 3.2.3. Quantitative Comparison and Discussion

As summarized in Figure 11, the RMSE, MAE, and MAPE values increase monotonically as the forecasting horizon extends, mainly due to the accumulation of temporal uncertainty and the weakening of correlations between distant time steps.

Across all horizons, the errors for cross-cabinet prediction are consistently higher than those for the same cabinet, attributed to differences in load profiles and environmental variability. Notably, the 6 h forecast yields the lowest errors and exhibits the most compact error distribution, consistent with its superior fitting performance.

Considering the balance between accuracy, stability, and generalization, subsequent applications adopt the 6 h forecast as the baseline task to ensure timely and reliable prediction in operational decision-making.

### 3.3. Performance Evaluation of the Lightweight LSTM Model

#### 3.3.1. Accuracy Comparison

In the 6 h forecasting task, the comparison of error metrics between the original and the lightweight LSTM models is presented in Table 1. The original LSTM achieved RMSE, MAE, and MAPE values of 0.10 °C, 0.08 °C, and 0.26%, respectively; while the lightweight LSTM recorded RMSE, MAE, and MAPE values of 0.27 °C, 0.21 °C, and 0.66%, respectively. A small expected increase in error was observed after quantization, but the overall values remained within the range considered acceptable for engineering applications. Combined with the substantial reductions in latency and resource consumption on the edge platform, the proposed scheme achieves a reasonable trade-off between accuracy and computational efficiency [25].

**Figure 12:**
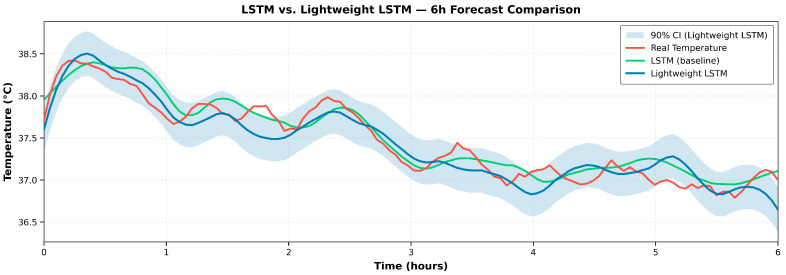
Comparison chart of fitting before and after lightweight.

To visually demonstrate the impact of quantization on the model’s fitting capability, Figure 12 presents a comparison between the measured temperature profile, the original LSTM prediction, and the lightweight LSTM prediction over a 6 h task, with confidence bands plotted around the two predicted curves. Both models capture the overall temperature trend and short-term fluctuations with high consistency. The lightweight model exhibits slight amplitude deviations at several peaks and troughs, and its confidence band is marginally wider; nevertheless, it remains well aligned with the measured curve. These results indicate that mixed-precision quantization markedly enhances the feasibility of edge deployment without causing a substantial loss in the ability to fit key temporal features [26].

By integrating the consistent conclusions drawn from both the error metrics and the fitting visualizations and considering the timeliness requirements of online operations and maintenance, a 6 h prediction horizon was adopted for subsequent experiments and applications. Within this horizon, the mixed-precision quantized LSTM can operate stably on resource-constrained platforms while maintaining the effective characterization of the key features of temperature evolution.

#### 3.3.2. Inference Efficiency

Five key metrics (Table 2) were selected to evaluate the lightweight model’s performance on a real edge-computing device.

**Figure 13:**
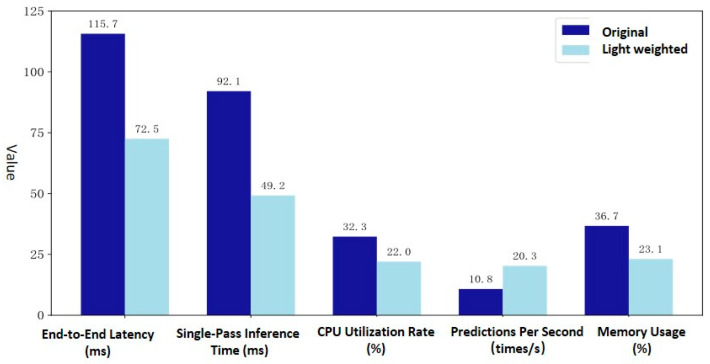
Performance evaluation of lightweight LSTM deployment on edge devices.

The experimental data were recorded using Python (3.10) libraries (time, psutil) and the official Raspberry Pi tool (PiDoc). The experimental results (as shown in Figure 13) demonstrate that the lightweight LSTM model outperforms the original model across all five evaluated metrics. Specifically, the inference speed is roughly doubled, achieving real-time prediction in the microsecond range. This enables the possibility of utilizing a single edge device for the simultaneous temperature prediction of multiple electrical joints.

#### 3.3.3. Resource Utilization and Discussion

Furthermore, the lightweight model exhibits significantly lower CPU and memory utilization compared to its predecessor. This resource saving is crucial, allowing the edge computing device to concurrently perform other vital computational tasks.

## 4. Discussion

The experimental results of this study demonstrate that the LSTM-based joint temperature-rise prediction approach achieves high accuracy and good stability in short-term forecasting tasks. Even under transfer deployment across different distribution cabinet joints and edge-computing platforms, the model consistently captures the temperature-variation trends effectively. Nevertheless, a comprehensive analysis of both the data and the deployment outcomes has also revealed several issues and limitations that warrant further investigation.

First, in cross-distribution cabinet joint scenarios, the prediction accuracy deteriorates markedly over longer horizons. For the 6 h prediction task, the RMSE and MAPE values are comparable to those in the same cabinet scenario; however, in the 24 h and 3-day predictions, the error growth in the cross-cabinet case is substantially higher than that in the same cabinet case. This discrepancy suggests that the model’s generalization capability for long-term forecasting is constrained when confronted with inter-equipment differences, variations in load patterns, and disturbances from environmental factors. Similar phenomena have been reported in other load-forecasting studies based on recurrent neural networks, indicating that this limitation is likely to be a common challenge.

Second, although the mixed-precision quantization scheme substantially reduces the model size and inference latency, the neuron connections operating under lower numerical precision exhibit heightened sensitivity to extreme operating conditions. The experiments revealed that when the temperature profile contains sharp peaks or rapid transitions, the lightweight LSTM shows slight deviations in peak amplitude and phase response compared with the full-precision model. This behavior is likely associated with the reduced numerical resolution introduced by quantization, and the effect becomes more pronounced in long-horizon forecasting. Therefore, for stringent condition monitoring and early-warning tasks under extreme states, the model requires accompanying precision compensation mechanisms—such as hybrid calibration or error-correction net-works—when operating at reduced precision.

Third, although the experimental data encompass real operating conditions from different distribution cabinets, the sample distribution remains imbalanced, particularly with a limited number of instances representing severe faults and high-temperature anomalies. This imbalance constrains the validation of the model’s stability under extreme operating patterns and may introduce bias when extrapolating certain performance evaluation results to broader field conditions. Therefore, future research should focus on expanding the sample space by increasing the collection of extreme-condition data or employing approaches such as simulated-fault experiments, thereby enhancing the model’s robustness across diverse operating states.

Finally, although the lightweight deployment on the Raspberry Pi platform has achieved the goal of real-time prediction, its performance in multi-task parallel processing and long-term operational stability remains to be fully assessed. In practical power distribution network operations, edge devices often need to run multiple monitoring and control tasks simultaneously, where factors such as computational resource allocation, memory management, and power-consumption strategies can all affect the sustained performance of the prediction module. This highlights the need for future work to explore more efficient model architectures (e.g., structured sparsity, convolution–recurrent hybrid networks) and hardware acceleration solutions (e.g., FPGA–NPU combinations) to further enhance the adaptability of the deployment environment.

## 5. Conclusions

In this study, a lightweight edge-enabled LSTM model was proposed for the short-term temperature prediction of electrical joints in low-voltage distribution cabinets. By integrating a self-developed multi-sensor acquisition system and an edge computing framework, the proposed model effectively achieved real-time temperature prediction with high accuracy and low computational cost. Comparative experiments demonstrated that the lightweight LSTM maintained a prediction accuracy comparable to the standard model, while significantly improving the inference efficiency and reducing the memory usage. These findings demonstrate the feasibility and engineering applicability of deploying a specific AI-based model in low-voltage distributed power systems, which have limited computing resources.

In future work, several directions could be explored to further enhance the performance and adaptability of the proposed framework. First, adaptive or transfer learning techniques can be introduced to improve the generalization across different cabinet types and environmental conditions. Second, multi-source data fusion involving humidity, vibration, and contact resistance need to be investigated to improve early fault detection accuracy. Finally, deployment strategies for large-scale edge networks and online incremental learning may be studied to achieve continuous model evolution and intelligent decision support for predictive maintenance in electrical distribution systems.:

## Figures and Tables

**Table 1:** Comparison of performance before and after model lightweighting.

Model	RMSE (°C)	MAE (°C)	MAPE (°C)
Original LSTM	0.10	0.08	0.26%
Lightweight LSTM	0.27	0.21	0.67%

**Table 2:** Key metrics to evaluate model performance running on the device.

Metrics	Unit	Definition
End-to-End Latency	ms	Total Time from Input Buffer to Prediction Output
Single-Pass Inference	ms	Average Time for a Single Prediction
CPU Utilization Rate	%	Average CPU Utilization during Model Execution on Raspberry Pi-5
Throughput	Times/s	Prediction Per Second
Memory usage	%	Percentage of Memory Utilization during Model Computation (Total RAM: 8 GB)

## Data Availability

The original contributions presented in this study are included in the article. Further inquiries can be directed to the corresponding author.

## Supplementary Materials

The following supporting information can be downloaded at: https://www.mdpi.com/article/10.3390/s25226816/s1, Figure S1: Temperature and humidity sensor for ambient environment in cabinet; Figure S2. Sensor of load current with PCB layout and transformer; Figure S3. Edge computation device; Figure S4. Experimental setup in the laboratory.

## Abbreviations

The following abbreviations are used in this manuscript:

ARIMA	Autoregressive integrated moving average
GRU	Gated recurrent unit
LSTM	Long short-term memory
RMSE	Root mean square error
MAE	Mean absolute error
MAPE	Mean absolute percentage error
RNN	Recurrent neural network
RMS	Root mean square
*μ*	Mean of the sequence
*σ*	Standard deviation of the sequence
FP32	Full-precision floating point data
INT8	8-bit integer
FP16	Half-precision floating point data
*S*	Scaling factor
*Z*	Zero point
