# Supplementary material for: An Edge-Enabled Lightweight LSTM for the Temperature Prediction of Electrical Joints in Low-Voltage Distribution Cabinets
Source: Sensors (Basel). 2025 Nov 7;25(22):6816. doi: 10.3390/s25226816 (PMC12655914; doi:10.3390/s25226816)
Supplement: Supplementary file 1 [file sensors-25-06816-s001.zip › sensors-3943181-supplementary.pdf]

**Supplementary Materials:**

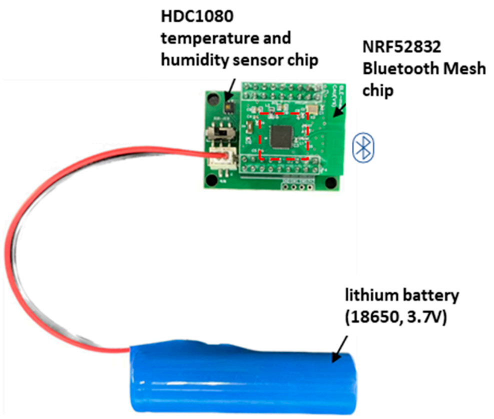


**Figure S1.** Temperature and humidity sensor for ambient environment in cabinet

Shown as Figure S1, The HDC1080 integrated circuit, employed as a temperature and humidity sensor, transmits its measurement data via Bluetooth Mesh to edge computing devices. The system is powered by a single 18650 lithium-ion battery, ensuring compactness and extended operational lifetime suitable for low-power wireless sensing applications.

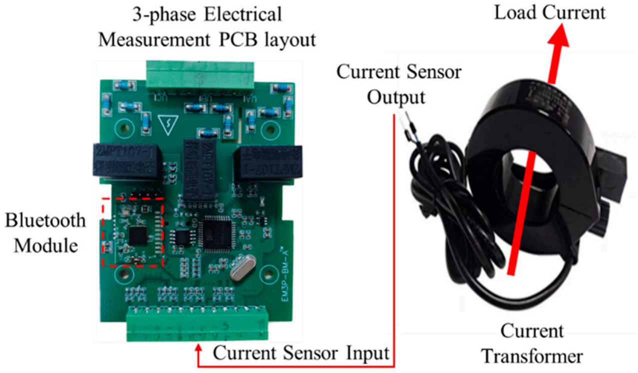


**Figure S2.** Sensor of load current with PCB layout and transformer

Shown as Figure S2, the current transformer converts high primary currents into low-level analog signals, which are then fed into a custom-developed three-phase energy metering module. This module subsequently transmits the acquired measurement data to an edge computing device via Bluetooth Mesh networking.

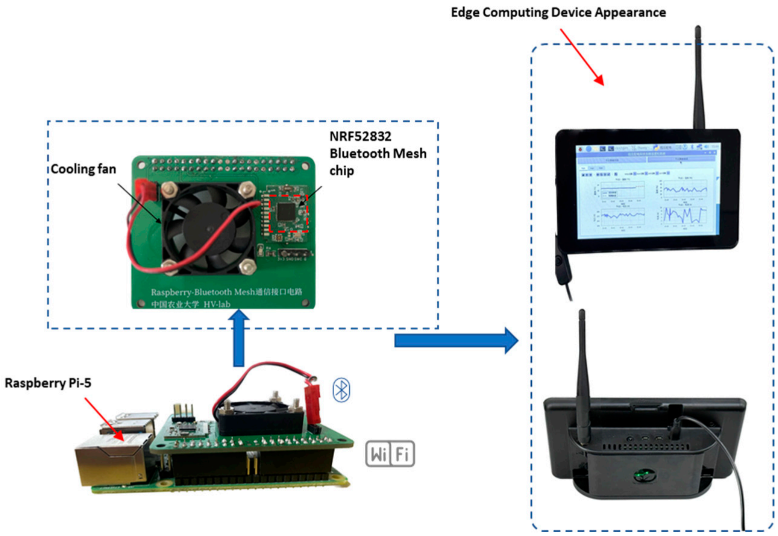


**Figure S3.** Edge computation device

Shown as Figure S3, an edge computing device based on the Raspberry Pi is developed to collect sensor data via Bluetooth Mesh communication. The acquired data undergoes preprocessing before being stored locally. In this work, a Long Short-Term Memory (LSTM)-based joint temperature prediction model is developed and deployed onto the edge computing device. The system is further equipped with an external touchscreen interface to enable user interaction and operational control.

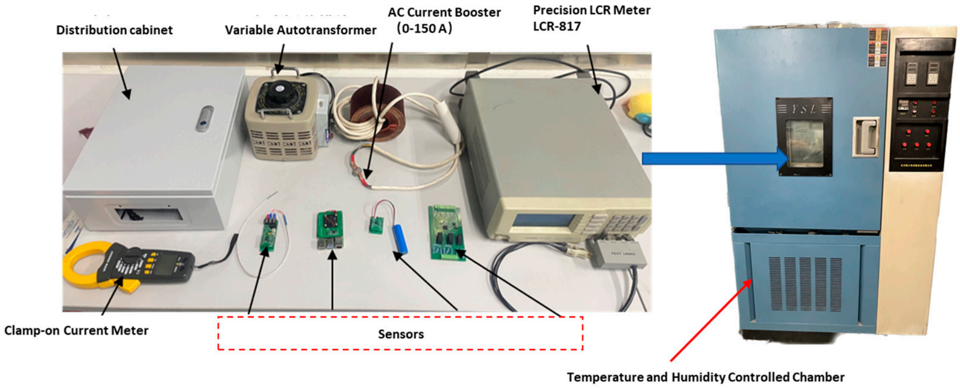


**Figure S4.** Experimental setup in laboratory

Shown as Figure S4, the temperature and humidity control chamber shown on the right side of the figure is employed to emulate the ambient environment surrounding a power distribution cabinet. The photograph illustrates the physical setup of the apparatus corresponding to Figure 4a in the main text.
